# Irradiation Haematopoiesis Recovery Orchestrated by IL-12/IL-12Rβ1/TYK2/STAT3-Initiated Osteogenic Differentiation of Mouse Bone Marrow-Derived Mesenchymal Stem Cells

**DOI:** 10.3389/fcell.2021.729293

**Published:** 2021-09-03

**Authors:** Fengjie Li, Rong Zhang, Changpeng Hu, Qian Ran, Yang Xiang, Lixin Xiang, Li Chen, Yang Yang, Shengwen Calvin Li, Gang Zhang, Zhongjun Li

**Affiliations:** ^1^Department of Blood Transfusion, The Irradiation Biology Laboratory, The Second Affiliated Hospital, The Third Military Medical University, Chongqing, China; ^2^Department of Pharmacy, The Second Affiliated Hospital, The Third Military Medical University, Chongqing, China; ^3^CHOC Children’s Research Institute, Children’s Hospital of Orange County, University of California, Irvine, Irvine, CA, United States; ^4^Department of Oral and Maxillofacial Surgery, The Second Affiliated Hospital, The Third Military Medical University, Chongqing, China

**Keywords:** irradiation, haematopoiesis, recovery, osteogenesis, IL-12

## Abstract

**Purpose:**

Repairing the irradiation-induced osteogenic differentiation injury of bone marrow mesenchymal stem cells (BM-MSCs) is beneficial to recovering haematopoiesis injury in radiotherapy; however, its mechanism is elusive. Our study aimed to help meet the needs of understanding the effects of radiotherapy on BM-MSC osteogenic potential.

**Methods and Materials:**

Balb/c mice and the BM-MSCs were used to evaluate the irradiation-induced osteogenic differentiation injury *in vivo*. The cellular and molecular characterization were applied to determine the mechanism for recovery of irradiation-derived haematopoiesis injuries.

**Results:**

We report a functional role of IL-12 in acute irradiation hematopoietic injury recovery and intend to dissect the possible mechanisms through BM-MSC, other than the direct effect of IL-12 on hematopoietic stem and progenitor cells (HSPCs). Specifically, we show that early use of IL-12 enhanced the osteogenic differentiation of BM-MSCs through IL-12Rβ1/TYK2/STAT3 signaling; furthermore, IL-12 induced osteogenesis facilitated bone formation and irradiation hematopoiesis recovery when transplanted BM-MSCs in the femur of Balb/c mice. For the mechanism of action, we found that IL-12 receptor beta 1 (IL-12Rβ1) expression of irradiated BM-MSCs was upregulated rapidly, coincidentally consistent with early use of IL-12 induced osteogenic differentiation enhancement. IL-12Rβ1 and tyrosine kinase 2 gene (Tyk2) silencing experiments and phosphotyrosine of signal transducer and activator of transcription 3 (p-STAT3) suppression experiments indicated the IL-12Rβ1/TYK2/STAT3 signaling was essential in IL-12-induced osteogenic differentiation enhancement of BM-MSCs.

**Conclusion:**

These findings suggested that IL-12 may exert BM-MSCs-based hematopoietic recovery by repairing osteogenic differentiation abilities damages through IL-12Rβ1/TYK2/STAT3 signaling pathway post-irradiation.

## Introduction

Radiotherapy is considered one of the most accepted and widely used medical treatments for tumors and cancers. However, even the lowest dose of localized irradiation exposure can result in adverse complications to adjacent normal organs, tissues, especially irradiation-sensitive organizations (Costa and Reagan, 2019). Irradiation causes myelosuppression and hematopoietic injuries, typical side effects, and patient radiotherapy obstacles (Greenberger and Epperly, 2009; Xu et al., 2011; Zhang et al., 2015; Seshadri and Qu, 2016; Zhao and Liu, 2016).

Hematopoietic injuries in irradiation include the decline of hematopoietic stem and progenitor cells (HSPCs) and the hemopoietic microenvironment (HM) damages, consisting of stromal cells regulating the growth and development of HSPCs (Costa and Reagan, 2019). Currently, hematopoietic injuries are commonly treated by hematopoietic stem cell transplantation (HSCT) in the clinic. However, HSCT’s curative effects are far from satisfactory. No long-term survivors have been reported in those who received current HSCT for severe irradiation damage of the bone marrow, even their temporary autologous blood cell recovery was observed. In such cases, the causes of death involved a wide potential exacerbation of hematopoietic and non-hematopoietic tissue injuries caused by the conditioning irradiation pretreatment. Therefore, the protection support system strategies, including combined transplantation with immunomodulatory drugs and mesenchymal stem cells, combined with HSCT, reached increasing attention. Hence, preserving and promoting HM biologic function became the target of hematopoietic reconstitution and rescuing hematopoietic injury (Moore, 2004; Greenberger and Epperly, 2009; Seshadri and Qu, 2016).

Bone marrow mesenchymal stem cells (BM-MSCs) are typical and essential components and regulators of the bone marrow haematopoiesis (Zhu and Emerson, 2004; Xu et al., 2011; Zhao and Liu, 2016). Their osteogenic differentiation abilities made their physical supporting role and a vital source of osteoblasts (Calvi et al., 2003), which directly constitute HM’s physical support and regulate its size and activity and offer haematopoiesis supporting functions (Calvi et al., 2003; Zhang et al., 2003; Muguruma et al., 2006). In irradiation, BM-MSCs also have more radioresistance than HSPCs (Sugrue et al., 2013; Zhang et al., 2015), but their osteoblasts differentiation potential was decreased, which subsequently crippled their haematopoiesis-supporting efficacy (Tohma et al., 2012; Sugrue et al., 2013; Wang et al., 2016).

IL-12, produced by monocytes, macrophages, and dendritic cells after challenge with bacteria or their products, has various effects; including anticancer, anti-infection, and clinical treatment of autoimmune diseases (Kobayashi et al., 1989; Colombo and Trinchieri, 2002; Darlak et al., 2014). In irradiation protection and treatment, IL-12 has been discovered as a potent cytokine in acute irradiation hematopoietic injury recovery, including increasing the number and the size of peripheral blood and hemopoietic cells (Chen et al., 2007; Basile et al., 2012; Gluzman-Poltorak et al., 2014; Gokhale et al., 2014; Gerber et al., 2015). Although these studies have found IL-12 may, directly and indirectly, promote HSPCs growth (Klein, 2004; Calvi, 2006; Chen et al., 2007), the mechanism of how it acts on HSPCs and HM to assist, haematopoiesis recovery remain to be clarified. The mechanism of how it affects on HSPCs and HM to help haematopoiesis recovery remains to be clarified.

In the present study, we focused on the effects and possible mechanisms of IL-12 on the osteogenic differentiation potential of BM-MSCs and these effects and mechanisms related to IL-12-induced irradiation hematopoietic recovery. We first found a single dose of IL-12 has direct remarkable osteogenesis promotion *in vivo* and *in vitro*. For that, the IL-12Rβ1/TYK2/STAT3 signal plays an indispensable role, especially in the IL-12 induced early osteogenic differentiation signal. Besides that, we first observed a direct positive correlation between osteogenic differentiation of BM-MSCs, bone formation of the femur, and haematopoiesis injury recovery in IL-12 treated groups. These findings furthered our understanding of haematopoiesis injury recovery effects of IL-12 in irradiation. They might provide a potential target of rescuing differentiation bias of BM-MSCs to promote hematopoietic support in radiotherapy.

## Results

### IL-12 Promotes the Haematopoiesis Recovery

**FIGURE 1:**
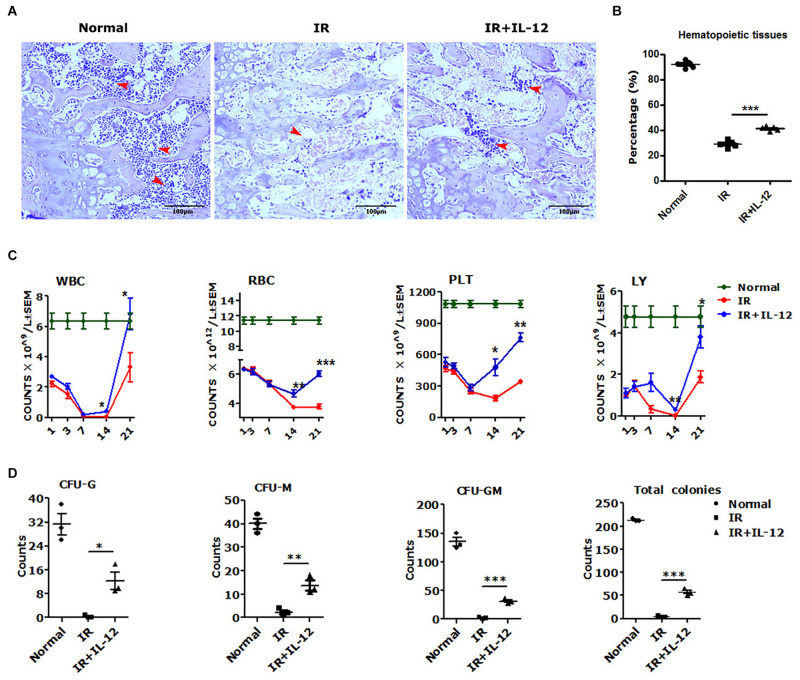
IL-12 promoted the haematopoiesis recovery in 5 Gy irradiated mice. **(A)** Hematoxylin and eosin (HE) staining of irradiated and subcutaneous femurs injected with a single dose of 1 ng/g IL-12 mice. The magnifications are 200×. Hematopoietic tissues were shown at the arrows (scale bar = 100 μm). **(B)** The percentage of hematopoietic tissues of the femoral bone tissue sections of irradiated and injected with a single dose of 1 ng/g IL-12 mice. **(C,D)** The peripheral blood cells (PBCs) count and the hematopoietic stem and progenitor cells (HSPCs) colonies of irradiated and injected with a single dose of 1 ng/g IL-12 mice. The results were considered significant at *P < 0.05, **P < 0.01 and ***P < 0.001.

To test the hematopoiesis effects of IL-12, we explored the bone marrow pathological, HSPCs colony formations, and peripheral blood cells (PBCs) profiles in irradiated mice, which were then promptly treated with a single dose of 1 μg/Kg IL-12 within 1 h after irradiation through subcutaneous injection. As shown in Figure 1A, the structure and the cellularity of femurs from irradiated mice, both treated with or without IL-12, were severely damaged on day 14 after irradiation. But IL-12 treated mice showed significantly more hematopoietic tissues in femurs (Figure 1B).

The PBCs in all irradiated mice took on a firstly decreasing, and then upon recovery increasing change. But the IL-12 treated mice performed better profiles in the blood cell change process. During the first declining phase, in IL-12 treated mice, PBCs decreasing attenuated significantly, and during the later recovery phase, approximately day 14 after irradiation, PBCs increasing accelerated greatly (Figure 1C). The HSPCs colony formation, detected on day 3 after irradiation, was also decreased in all irradiated mice, but in IL-12 treated mice, significantly more colonies of CFU-G, CFU-M, CFU-GM, and total colony-forming units (CFU) were reproduced (Figure 1D and Supplementary Figure 1).

### IL-12 Promotes Osteogenesis *in vivo* and *in vitro*

**FIGURE 2:**
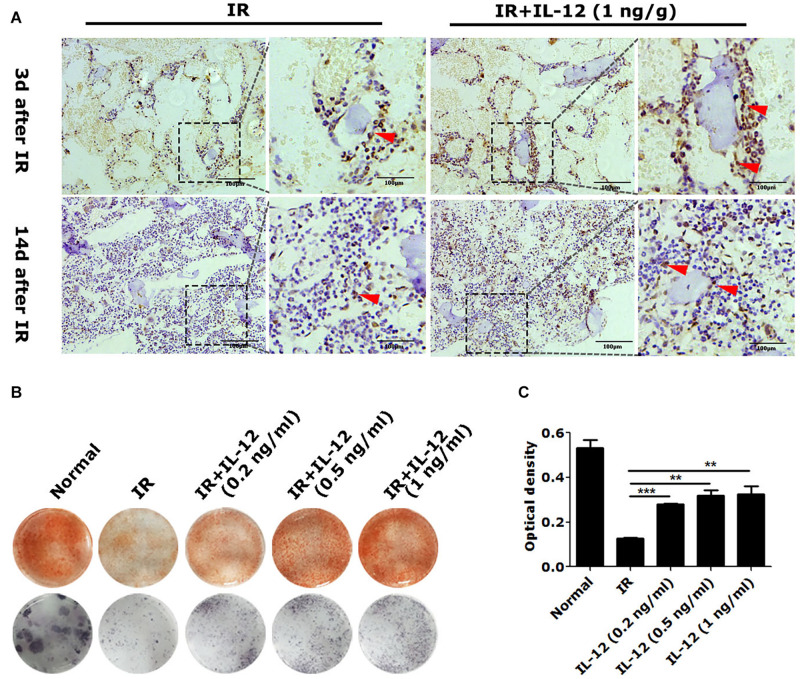
The osteogenesis effects of IL-12 in 5 Gy irradiated mice and 9 Gy irradiated bone marrow mesenchymal stem cells (BM-MSCs). **(A)** Immunohistochemistry staining of femur sections and osteoblasts at the arrow (scale bar = 100 μm). **(B)** Alizarin red S staining of cells calcium depositions and the ALP activities staining of osteoblasts differentiated cells in irradiated BM-MSCs co-cultured with 0.2, 0.5, and 1 ng/ml IL- 12 osteogenesis inducing medium for 14 days. The magnifications are 200×. **(C)** Quantification of calcium depositions using assays of colorimetric determination of the dye extraction in cells stained with alizarin red. The determination absorption wavelength was 562 nm. All the data were presented as the mean ± SEM, *n* = 3, The results were considered significant at **P < 0.01 and ***P < 0.001.

We next examined the effect of IL-12 on osteogenesis *in vivo*. Results showed osteogenic tissue around the endosteum of cancellous femurs of IL-12 treated mice was more than that of without IL-12 treated ones (Figure 2A). *In vitro*, the committed osteogenic differentiation of irradiated BM-MSCs co-cultured with IL-12 was also significantly increased. Besides that, IL-12 induced osteogenesis enhancing showed sufficient concentrations of 0.2–1 ng/ml (Figures 2B,C). These results indicated an apparent promotion of osteogenesis after irradiation.

### IL-12Rβ1 Plays a Crucial Signal in IL-12 Induced Early Osteogenesis of BM-MSCs After Irradiation

**FIGURE 3:**
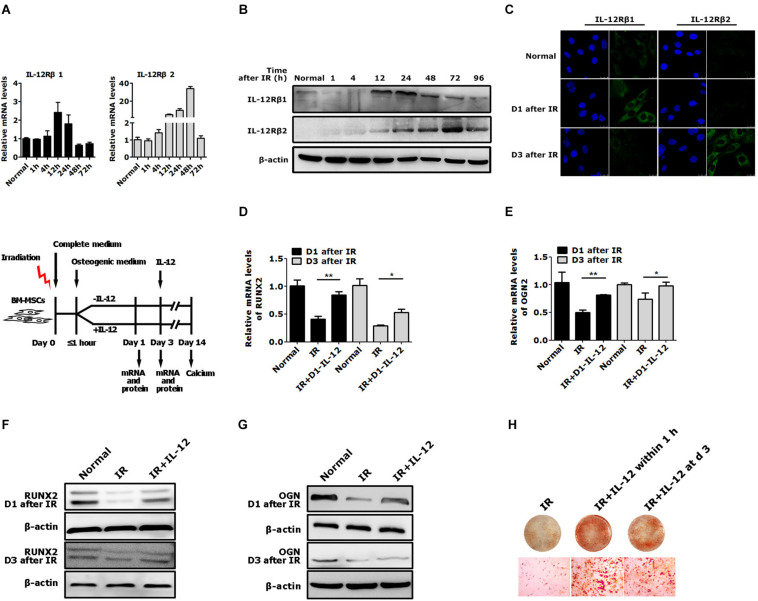
The expression of IL-12 receptors and the osteogenic process of BM-MSCs co-cultured with 0.2 ng/ml IL-12 in osteogenesis inducing medium after irradiation. **(A)** RT-PCR, **(B)** Western blot, **(C)** immunofluorescence analysis of expressions of IL-12Rβ1 and IL-12Rβ2 **(D–G)** The mRNA and protein levels of Runx2 and Ogn in irradiated cells co-cultured with IL-12 in osteogenesis inducing medium for 1 and 3 days. Three replicates were used in the mRNA levels analysis, and data were presented as the mean ± SEM. The results were considered significant at *P < 0.05 and **P < 0.01. **(H)** Alizarin red S staining of calcium depositions in cells co-cultured IL-12 on day 1 and day 3 after irradiation in osteogenesis inducing medium for 14 days. The magnifications are 200×.

Considering IL-12 biological activities depended on its receptors, IL-12Rβ1 and IL-12Rβ2, they and their corresponding JAK/STATs were estimated in irradiation conditions. After irradiation, both IL-12Rβ1, IL-12Rβ2 expressions increased at first and then decreased. It was worth noting that IL-12Rβ1 expression was swifter than IL-12Rβ2, mainly manifested in the fact that the RNA expression peak of IL-12Rβ appeared at 12 h after irradiation. The protein expression peak occurred 24 h after irradiation. In contrast, the protein expression peaks of IL-12Rβ2 appeared at 24 and 72 h after irradiation, respectively (Figures 3A–C).

Based on the expression profiles of the IL-12Rβ1 and IL-12Rβ2 after irradiation, we tried to figure out whether they correlated to IL-12-induced osteogenesis. We designed two experiments. First, irradiated cells were co-cultured with IL-12, while IL-12 was administrated within 1 h after irradiation. The osteogenic genes were then estimated at day 1 and day 3 after co-culturing. Second, we investigated the time-dependent effects of IL-12 on osteogenesis by analyzing the osteoblastic differentiation of irradiated cells co-cultured with IL-12 within 1 h and on day 3 after irradiation. According to the results, in the first 3 days of co-cultivation, the expression of Runx2 and Ogn increased significantly when IL-12 was administered within 1 h after irradiation – their expression was more pronounced in cells obtained on day 1 (Figures 3D–G). Besides that, IL-12 administrated within 1 h after irradiation-induced more matrix mineralization in BM-MSCs than that on day 3 (Figure 3H), suggesting that the earlier the administration of IL-12, the better osteogenesis would be brought on; and it seemed that IL-12 played an essential role in the early stage of the osteogenesis process in BM-MSCs. These results not only confirmed IL-12-induced osteogenic enhancement of BM-MSCs but also suggested a potential role of IL-12Rβ1 in IL-12 induced osteogenic differentiation in irradiation.

### The Necessity of IL-12Rβ1/TYK2/STAT3 Signaling in IL-12-Induced Osteogenesis

**FIGURE 4:**
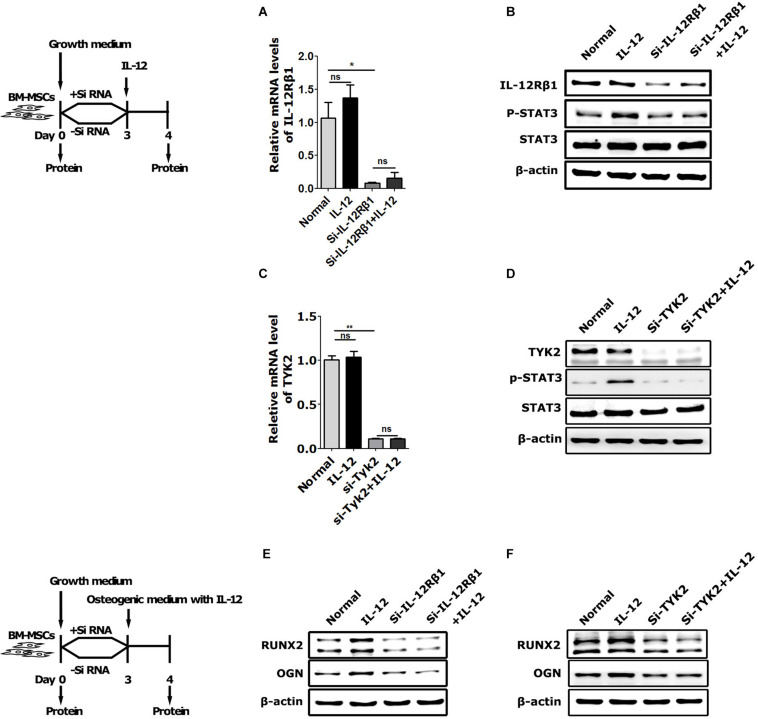
The role of IL-12Rβ1/TYK2 signal in IL-12-induced osteogenesis of BM-MSCs. **(A–D)** qRT-PCR analysis and western blot analysis of expressions of IL-12Rβ1, STAT3, and p- STAT3 in IL-12Rβ1 and TYK2 siRNA transfected cells co-cultured in complete media with or without 0.2 ng/ml IL-12 for 24 h. All the data were presented as the mean ± SEM, *n* = 3. The results were considered no significant at ns, and significant at *P < 0.05 and **P < 0.01. **(E,F)** Western blot analysis of expressions of Runx2, Ogn in IL-12Rβ1, and Tyk2 siRNA transfected co-cultured in osteogenesis inducing medium with or without 0.2 ng/ml IL-12 for 24 h. The cells without transfection were cultured without IL-12 used as controls.

To further elucidate the IL-12Rβ1 signaling function in IL-12-induced early osteogenesis, the main downstream effectors – IL-12Rβ1, TYK2, and STAT3 – were evaluated. Specifically, the cells transfected with Small interfering RNA (siRNA) for 72 h were co-cultured with IL-12 for 24 h. Results showed that p-STAT3 activities and Runx2 and Ogn expressions were promoted in IL-12 treated cells, abrogated by the specific siRNA (Figure 4).

**FIGURE 5:**
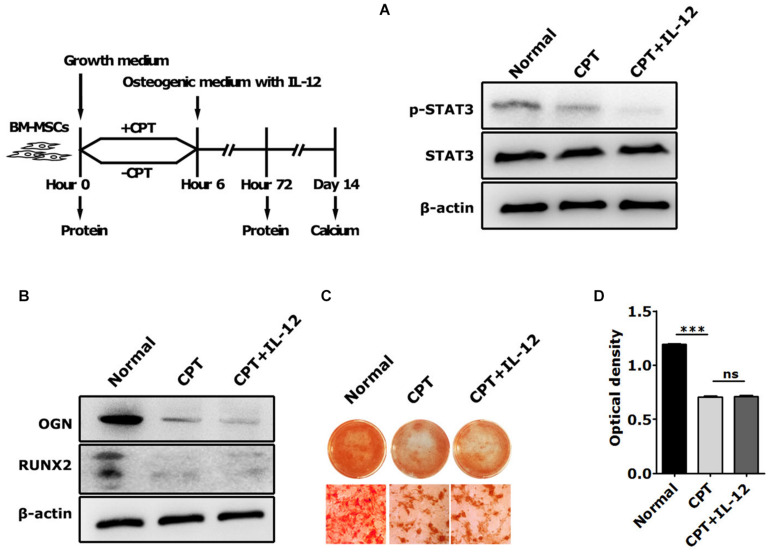
The role of STAT3 activation in IL-12-induced osteogenesis of BM-MSCs. **(A)** Western blot analysis of STAT3, p-STAT3, and Tyk2 expressions in CPT treated cells co-cultured in osteogenesis inducing medium with or without 0.2 ng/ml IL-12 for 24 h **(B)** western blot analysis of Runx2, Ogn, Tyk2 expressions in CPT treated cells co-cultured in osteogenesis inducing medium with or without 0.2 ng/ml IL-12 for 24 h **(C,D)** alizarin red S staining of calcium depositions, the magnifications are 200×, and quantification of calcium depositions using the assay of colorimetric determination of the dye extraction in cells stained with by alizarin red. The determination absorption wavelength was 562 nm. All the data were presented as the mean ± SEM, *n* = 3. The results were considered no significant at ns, and were considered significant at ***P < 0.001.

Next, we used cryptotanshinone (CPT), a unique inhibitor of STAT3, to significantly inhibit the activation of STAT3 by selectively blocking the Tyr705 phosphorylation of STAT3 and the dimerization of STAT3. In coincidence with the results of interfering with IL-12 Rβ1 and TYK2, the activation increasing of p-STAT3 and Runx2 expressions of Runx2, Ogn in IL-12 co-cultured cells were also significantly abrogated (Figures 5A,B). Furthermore, the osteogenesis enhancement that occurred in IL-12-co-cultured cells did not re-occur in CPT-treated cells (Figures 5C,D). These results indicated that the inhibition of STAT3 directly impaired the IL-12- induced osteogenesis.

### IL-12 Induced Osteogenesis Facilitated the Bone Formation and the Irradiation Haematopoiesis Recovery

**FIGURE 6:**
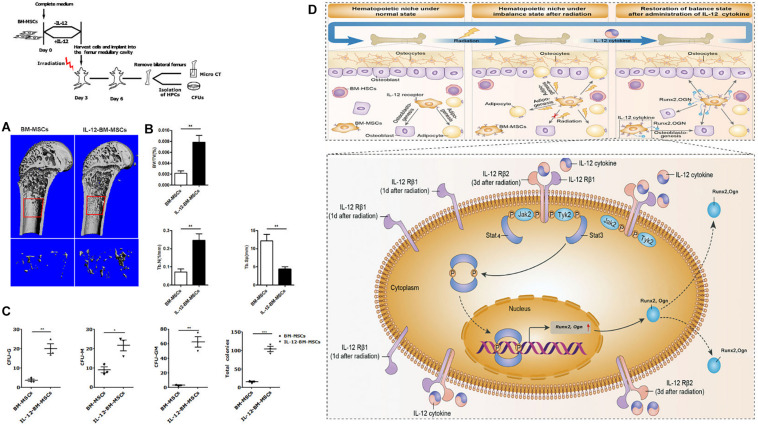
The haematopoiesis supports bone formations of BM-MSCs co-cultured with 0.2 ng/ml IL-12 for 24 h and proposed a mechanism of IL-12-induced osteogenesis in BM-MSCs after irradiation. **(A,B)** Bone mineral density analysis of bilateral femur of 5 Gy irradiated mice which were cells transplanted at the bilateral femur by using Scanco Medical CT-40 instruments. All the data were presented as the mean ± SEM, *n* = 5. **(C)** HSPCs colonies analysis bilateral femur of 5 Gy irradiated mice which were cells transplanted at the bilateral femurs. **(D)** Firstly, irradiation-induced the upregulation of IL-12 receptors, especially IL- 12Rβ1. Then the TYK2/STAT3 signaling activation, such as Tyk2 activation, STAT3 phosphorylation, nuclear translocation after dimerization, were triggered. In succession, the expression initial of osteogenic genes, Runx2 and Ogn, increased, promoting the osteoblast differentiation of BM-MSCs. ↑, increase. The results were considered significant at *P < 0.05, **P < 0.01, and ***P < 0.001.

Based on the fact that the better hematopoiesis recovery effect of IL-12 in irradiated mice could be obtained when IL-12 was administrated within 24 h after irradiation (21), we further estimated the bone formation and corresponding bone marrow hematopoiesis in the femur of irradiated mice transplanted with BM-MSCs which were co-cultured with IL-12 (IL-12-BM-MSCs) for 24 h. For this, we transplanted IL-12-BM-MSCs into the left femur and transplanted BM-MSCs, co-cultured without IL-12, into the right femur of the same individual irradiated mouse to exclude individual differences and to investigate the direct effect of cells on bone marrow hematopoiesis by minimally invasive surgery (Supplementary Figure 2a). As shown in Figures 6A,B, femur transplants with IL-12-BM- MSCs showed increased cancellous bone mass than those transplanted with BM-MSCs. Parallelly, HSPCs in femur transplanted with IL-12-BM-MSCs also had better colony-formations (Figure 6C and Supplementary Figure 2b).

We confirmed that IL-12 could promote irradiation haematopoiesis recovery *in vivo* and demonstrated its role in promoting the osteogenic differentiation in irradiated BM-MSCs. For the latter, we are sure that IL-12Rβ1 and its related signals, TYK2 and STAT3, played an important role, at least in the early period after irradiation. To this end, we proposed the possible mechanism of IL-12 induced osteogenic differentiation in BM-MSCs after irradiation. Irradiation induced the swift upregulation of IL-12 receptors, especially IL-12Rβ1. At this time, IL-12 triggered the downstream TYK2/STAT3 signaling and increased the STAT3 phosphorylation, which then increased its dimerization and nuclear translocation. Lastly, the osteogenic gene expressions were promoted, and the osteogenesis of BM-MSCs was enhanced, and at the same time, the hematopoiesis recovery happened (Figure 6D).

## Discussion

As a multifunctional cytokine, IL-12 bioactivities are involved in the proliferation, differentiation, survival, and apoptosis of various cell types (Watford et al., 2004; Nicolaidou et al., 2012; Gerber et al., 2015). What appeals to us is the considerable hematopoietic prevention and recovery functions in acute irradiation-induced IL-12, making it a brilliant and promising application in irradiation protection (Kerkar and Restifo, 2012; Singh et al., 2015). Even IL-12 showed the ability to directly act on HSPCs to promote their proliferation and differentiation (Chiba et al., 2018), while the long-term hematopoietic stem cells (LTR-HSC) showed a lack of IL-12 receptors (Chen et al., 2007). Due to the irradiation sensitivity, the function of HSPCs was seriously damaged in acute irradiation; it seemed hard to explain the robust haematopoiesis recovery of IL-12 only through its direct action on HSPCs. With a supportive role in the HM (Abbuehl et al., 2017; Wu et al., 2018) and the relatively greater resistance to irradiation (Chen et al., 2006; Singh et al., 2012) of BM-MSCs, IL-12 might directly affect the HM and contribute to haematopoiesis facilitation of BM-MSCs by regulating osteoblasts’ differentiation potential of BM-MSCs, as evident in the literature.

Previous research indicated that IL-12 could conduct robust irradiation hematopoiesis recovery in mice following exposure to irradiation when it was administered 24 h before or within 1 h after irradiation exposure (Basile et al., 2012). Therefore, all IL-12 administration of animals or cells was conducted within 1-h post-irradiation unless otherwise specified. As expected, IL-12 did exert considerable haematopoiesis facilitation in the bone marrow and PBCs in irradiated mice 21-day post-irradiation. Besides that, IL-12 exhibited an apparent osteogenesis promotion *in vivo* and *in vitro* (see Figure 2). The significantly better colony-formation abilities were also observed in IL-12 treated irradiated mice. These results suggested IL-12 might have a positive role in maintaining the HM function, such as retaining the osteogenic differentiation function of BM-MSCs in irradiation.

IL-12 manifests its bioactivities through the receptors of IL-12Rβ1 and IL-12Rβ2 (Supplementary Figure 3), and their corresponding tyrosine kinases activate the signaling cascade. Two tyrosine kinases of IL-12 are TYK2 and JAK2, and they interact with IL-12Rβ1 and IL-12Rβ2, respectively. The subsequent STAT4 and STAT3 are then activated for further IL-12 signaling transduction (Watford et al., 2004; Nicolaidou et al., 2012; Feng et al., 2016). Generally, the IL-12Rβ2/JAK2/STAT4 pathway and its activated IFN-gamma play a significant role in the common bioactivities of IL-12. Simultaneously, no direct function of IL- 12Rβ1 appears to be found, except for the essential subunits responsible for binding to IL-12 (Weaver et al., 2015). But increasing research indicates that IL-12Rβ1, acting in single or aiding IL-12 signaling, has many unknown functions (Robinson, 2015).

Furthermore, the STAT3, one of the positive molecules in IL-12 biological signal, quickly activated by IL-12 signal in T cells, was not only proven to perform a pivotal role in maintaining host homeostasis, anti-tumors, and immunomodulation but also was continuously identified as having a strong relationship with osteoblast differentiation and bone formation; and can be activated by various cytokines, growth factors and other stimuli (Derecka et al., 2012; Feng et al., 2016). The activation of STAT3 upregulates osteogenic-related factors – ALP, BMP2, and RUNX2 (Itoh et al., 2006; Derecka et al., 2012; Xie et al., 2018). IL-12Rβ1 is rapidly upregulated in irradiated BM-MSCs, with the IL-12Rβ1 expression peak detected at 24-hour post-irradiation. The data shows that the better osteogenic effects IL-12-induces, the more osteogenic gene expression increases, and the more calcium nodules form in BM-MSCs. Thus, it is reasonable to speculate that the IL-12Rβ1/TYK2/STAT3 may have a crucial role in mediated IL-12-induced osteogenesis.

Next, we focused on the relationship between STAT3 activation and IL-12-induced osteogenic promotion. We estimated the activation of STAT3 in IL-12-BM-MSCs by interfering with its necessary upstream signals, IL-12Rβ1 and TYK2, and inhibiting its phosphorylation at tyrosine 705 by using a unique inhibitor (CPT). Interestingly, as predicted, BM-MSCs co-cultured with IL-12 showed a pronounced activation of STAT3; and interfering IL-12Rβ1 and TYK2 signals abrogated the phosphorylations of STAT3 and upregulation of osteogenic genes. IL-12-induced osteogenesis enhancement appeared directly related to phosphorylation of tyrosine 705 of STAT3 in BM-MSCs because significant ontogenesis was eliminated in CPT treated cells. It is worth noting that a short time period, 24 h, of co-culturing with IL-12 was used in all the interfering and inhibiting experiments, which means only IL-12Rβ1 was upregulated in irradiation. These findings suggested a crucial timeframe of IL-12-induced osteogenic promotion in irradiated BM-MSCs and confirmed a direct positive regulating relationship between IL-12Rβ1/TYK2/STAT3 signaling and IL-12-induced osteogenic differentiation in BM-MSCs.

At last, we examined in parallel the hematopoietic supporte of BM-MSCs co-cultured with IL-12 for 24 h (IL-12-BM-MSCs) by HSPCs colony-forming assays and the bone formation of IL-12-BM-MSCs transplanted irradiated mice by analyzing the bone mineral density of the femur. Results showed more apparent colonies and bone formations in IL-12- BM-MSCs transplanted mice. As we knew, good bone formation is directly beneficial to reduce the bone loss common in irradiation (Costa and Reagan, 2019) and is indirectly beneficial to the reconstruction of haematopoiesis. Although research is needed, these findings suggest the parallel relationship of the hematopoietic supporting and bone formative promotion of IL-12-BM-MSCs and further strengthen our hypothesis that IL-12 exerts its hematopoietic support through its bone mass enhancing functions on BM-MSCs.

The effects of IL-12 on osteogenesis promotion in BM-MSCs made it practical to research its haematopoiesis recovery effects and the biological functions of BM-MSCs in bone marrow, as both of them are far from clear (Zhang et al., 2021). We clarify that IL-12-regulated signal transduction plays different roles in BM-MSCs, either interfering with osteoclasts formation alone or associating with the other cytokines (Horwood et al., 2001; Kitaura et al., 2011; Yoshimatsu et al., 2015). Even though further research is needed in light of clonal evolution (Lee and Li, 2020), our present study provides the first dataset to illustrate that IL-12 exerts a direct enhancement on osteogenic differentiation of irradiated BM-MSCs through its IL-12Rβ1/TYK2/STAT3 signal pathway. These results shed new light on the protective role of IL-12 in haematopoiesis recovery after irradiation damage, thereby inspiring development of a new treatment strategy for irradiation-induced osteoporosis.

## Materials and Methods

### Animals

Male and female Balb/c mice, aged 4–6 weeks, were provided by the Experimental Animal Center and housed in a specific pathogen-free animal facility of the Laboratory Animal Centre. The Animal Experiment Ethics Committee approved all experiments in this study.

### Cell Culture

Balb/c mice BM-MSCs, complete medium containing, and osteogenesis inducing medium were purchased from Cyagen Biosciences Inc. Cells were cultured at 37°C under a 5% CO2 atmosphere. The culture medium was replaced every 2–3 days. Cells at passage 8 were used in subsequent experiments.

### Pre-conditioning Irradiation

For all irradiation procedures, both cells and animals were total bodies irradiated (TBI) with a single dose of ^60^Co-γ using an irradiation source (Zhang et al., 2015) with a source-surface distance of 150 cm and at a rate of 0.69 Gy/min at the Irradiation Research Centre. The dose for cells was 9 Gy, and for animals was 5 Gy.

### Reagents

The mouse IL-12 was purchased from BioLegend, San Diego, CA, United States. Small interfering RNA targeting mouse Tyk2 was chemically synthetic in RiboBio Co., Ltd., Guangzhou, China. Small interfering RNA targeting mouse IL-12Rβ1 was purchased from Santa Cruz Biotechnology Inc. The inhibitor of STAT3, CPT, was purchased from MedChemExpress. The bone marrow washing medium, StemSpan^TM^ SFEM culture medium, the methylcellulose-based medium, and the culture plates, SmartDish^TM^ plate, were purchased from Stemcell Technologies Inc., Canada.

The primary antibodies used for western blotting and immunohistochemical analysis were the following: anti-IL-12Rβ1 (Bioss), anti-IL-12Rβ2 (Bioss), anti-RUNX2 (Runt-related transcription factor 2, Santa Cruz Biotechnology Inc.), anti-Osteoglycin (Ogn, Santa Cruz Biotechnology Inc.), anti-Osterix (Abcam, ab22552) anti-β-actin (Santa Cruz Biotechnology Inc.), anti-phosphorylated STAT3 (Tyr705) mouse monoclonal (mAb) (Cell Signaling Technology), anti-STAT3 (124H6) mouse mAb (Cell Signaling Technology), rabbit anti-TYK2 antibody (Abnova, Taipei, Taiwan).

### Quantitative Polymerase Chain Reaction

Total RNA was isolated using Trizol reagent (Invitrogen). The first-strand cDNA was synthesized using a reverse transcription reagent kit (TOYOBO, FSK-100, Osaka, Japan), following the manufacturer’s instructions. Next, real-time qPCR (RT-qPCR) was performed in triplicate using FastStart Universal SYBR Green Master Mix (Roche, Germany). The data were analyzed using the ^ΔΔ*CT*^ method. Experiments were performed in triplicate.

The sequences of the primers used in RT-qPCR are as follows: β*-actin* forward, 5′-TGGAATCCTGTGGCATCCATGAAAC-3′; β*-actin* reverse, 5′-TAAAACGCAGCTCAGTAACAGTCCG-3′; *Runx2* forward, 5′-GTGCCCAGGCGTATTTCAGATG-3′; *Runx2* reverse, 5′-GCGGGGTGTAGGTAAAGGTCGC-3′; *Ogn* forward, 5′-GTGGTCACATGGATAGCCTTTAGTC-3′; *Ogn* reverse, 5′-GAGCATATTTAGTTTGTTTGGGTGA-3′; *IL-12R*β*1* forward, 5′ATGCGCTGGTGGTCGAGATGC-3′; *IL-12R*β*1* reverse, 5′CCCGGCTCCGCAGTCTTATG-3′; *IL-12R*β*2* forward, 5′CGACGCTCTCAAAACTCACATCC-3′; *IL-12R*β*2* reverse, 5′TTTGCCGGAAGTAACGAATTGAG-3′; *Tyk2* forward, 5′-GTGCCTTCCGTGTTCAGCGTGTG-3′; *Tyk2* reverse 5′-GCCCAGAACGAATAGACTCAGGAA-3′.

### Western Blotting

Total lysates proteins were extracted with cell lysis buffer (Beyotime, Shanghai, China) and were denatured by boiling. Next, the cell lysates were separated on 4–12% SDS- polyacrylamide gels and transferred onto polyvinylidene fluoride membranes (PVDF Western Blotting Membranes, Roche). The membranes were blocked in PBS buffer [50 mM Tris–HCl, 150 mM NaCl, and 0.1% Tween (pH 7.6)] supplemented with 5% non-fat dry milk incubated with the appropriate antibodies for 12 h. An HRP-labeled secondary antibody and a chemiluminescent detection system (Phototope-HRP western blot detection kit; New England Biolabs, Ipswich, MA, United States) were used for developing the blots.

### Hematopoiesis and Osteoblasts Detections

The peripheral blood cell counts were estimated by analyzing the eyeball blood with the automatic animal blood cell analyzer (Prandre XFA6030). For the hematopoiesis of bone marrow, the bilateral femur was removed and fixed in 10% formalin and embedded in paraffin wax for micro-sectioning at 5 μm and routine hematoxylin and eosin (HE) staining. All slides were examined under the microscope (Axio Imager 2, Zeiss, Germany). The volume of bone marrow hematopoietic tissues was detected by image analysis and reporting system (YC.YX-2050).

For bone marrow HSPCs colony assays, mice were euthanized by injecting 150 mg/kg sodium pentobarbital, and the bilateral femurs were removed. The bone marrows were aseptically flushed into a serum-free expansion medium (StemSpan^TM^ SFEM, StemCell Technologies Inc., Vancouver, BC, Canada) using a syringe fitted with a 2l-gauge needle. Cells were counted and cultured in methylcellulose media (MethoCult^TM^ GF, StemCell Technologies Inc., Vancouver, BC, Canada) following the manufacturer’s instruction. A total of 3,00,000 cells from unirradiated mice were used as normal control (Normal). The CFU of granulocyte (CFU-G) and macrophage (CFU-M), CFU-granulocyte macrophage (CFU-GM), CFU-granulocyte, erythroid, macrophage, megakaryocyte (CFU-GEMM) were analyzed according to the technical manual, mouse CFU assays using methoCult^TM^(Stemcell Technologies Inc., Canada) *in situ* by light microscope (Leica DMIRB, Germany).

### Osteogenic Differentiation and Bone Formation Detection of BM-MSCs

Bone marrow mesenchymal stem cells committed osteogenic differentiation detection were analyzed by mineralized matrix staining for calcium mineral deposits using an alizarin red S staining kit (Cyagen). ALP activities were tested using ALP staining complied with 5-bromo-4-chloro-3-indolyl phosphate and nitro-blue-tetrazolium (BCIP/NBT) alkaline phosphatase color development kit (Beyotime Biotechnology). The mineralized matrix was examined and photographed using a light microscope (Leica DMIRB, Germany). The bone mineral densities were analyzed at the bottom of the distal growth plate. The epiphyseal cap structure disappeared and continued for 95 slices (10.5 μm/slice, using Scanco Viva CT40) toward the femur’s proximal end.

### Cells Transplantation Assays

Before transplantations, irradiated mice were anesthetized with 20 μg/kg pentobarbital sodium, using a 10 μl microliter syringe purchased from Gaoge industrial and trading Co., Ltd. Subsequently, cells suspended in a minimal volume of 3 ml PBS and 10,000 cells were injected intrafemorally through the patellar surface. The whole-cell transplantations were carried out within 1 h after TBI.

### Transfection, Inhibition, and Immunofluorescence Assays

Briefly, cells transfected with special siRNA (final concentration, 50 nM) using Lipofectamine 3000 (Invitrogen, Carlsbad, CA, United States) follow the manufacturer’s instructions. After 72 h of transfection, cells were co-cultured with or without 0.2 ng/ml IL-12 for 24 h. For the inhibition of STAT3, the inhibitor was added in the culture medium at 50 nM for co-culturing 24 h, and then cells were co-cultured with or without 0.2 ng/ml IL-12 for 24 h. And then, all the cell samples were collected for further detection.

Cells for immunofluorescence tests were fixed with 4% formaldehyde. They were then incubated overnight at 4°C with a primary antibody and were incubated with FITC-labeled secondary antibody for 1 h at RT. After each step, the prepared specimens were counterstained with 5 μg/ml 4,6-diamidino-2-phenylindole and were observed with laser scanning confocal microscopy (LSCM) (Olympus, Japan).

### Statistical Analysis

All data were presented as the mean ± SEM. Statistical analysis was performed using GraphPad Prism version 6.0. An independent unpaired *t*-test was used to compare the experimental groups’ data with those obtained from the control group. The results were considered significant at ^∗^*P* < 0.05, ^∗∗^*P* < 0.01, and ^∗∗∗^*P* < 0.001.

## Data Availability Statement

The raw data supporting the conclusions of this article will be made available by the authors, without undue reservation.

## Author Contributions

FL and RZ contributed to conception and designing of the work, data analysis, and data interpretation. SL contributed to manuscript preparation, editing, and review. CH and YY performed parts of the data acquisition and analyses for cell culture. QR and YX conducted parts of the experiments and prepared parts of the graphs and figures. LC and LX conducted the immunofluorescence experiments. GZ and ZL participated in the study design and experimental design. All authors read and approved the final manuscript.

## Conflict of Interest

The authors declare that the research was conducted in the absence of any commercial or financial relationships that could be construed as a potential conflict of interest.

## Publisher’s Note

All claims expressed in this article are solely those of the authors and do not necessarily represent those of their affiliated organizations, or those of the publisher, the editors and the reviewers. Any product that may be evaluated in this article, or claim that may be made by its manufacturer, is not guaranteed or endorsed by the publisher.

## Supplementary Material

Supplementary Figure 1 IL-12 promotes hematopoietic stem and progenitor cells (HSPCs) colonies formation after irradiation. **(a)** HSPCs colonies without stain were taken photos under the light microscope. 2.5X. **(b)** Different types of colony-forming units (CFU) of granulocyte (CFU-G) and macrophage (CFU-M), CFU-granulocyte macrophage (CFU-GM), CFU-granulocyte, erythroid, macrophage, megakaryocyte (CFU-GEMM) were analyzed according to the technical manual.

Supplementary Figure 2 The capacity of bone marrow colony-forming unity fibroblast (CFU-F) in two femurs of the same individual after transplantation of IL-12 treated bone marrow mesenchymal stem cells (BM-MSCs) (IL-12-BM-MSCs) and BM-MSCs **(a)** Minimally invasive transplantation at distal femurs of irradiated mice and **(b)** HSPCs colonies of the left femurs, invasive transplantation with BM-MSCs treated with IL-12 for 24 h, and right femurs, invasive transplantation with BM-MSCs treated without IL-12, of the same individual without staining.

Supplementary Figure 3 The function of IL-12 receptors in the osteogenic process of BM-MSCs co-cultured with 0.2 ng/ml IL-12 in osteogenesis inducing medium after irradiation. Alizarin red S staining of calcium depositions in cells co-cultured IL-12 on day 1 after irradiation in osteogenesis inducing medium for 14 days. The magnifications are 200×.
