# Supplementary figures and images for: Irradiation Haematopoiesis Recovery Orchestrated by IL-12/IL-12Rβ1/TYK2/STAT3-Initiated Osteogenic Differentiation of Mouse Bone Marrow-Derived Mesenchymal Stem Cells
Source: Front Cell Dev Biol. 2021 Sep 3;9:729293. doi: 10.3389/fcell.2021.729293 (PMC8446663; doi:10.3389/fcell.2021.729293)

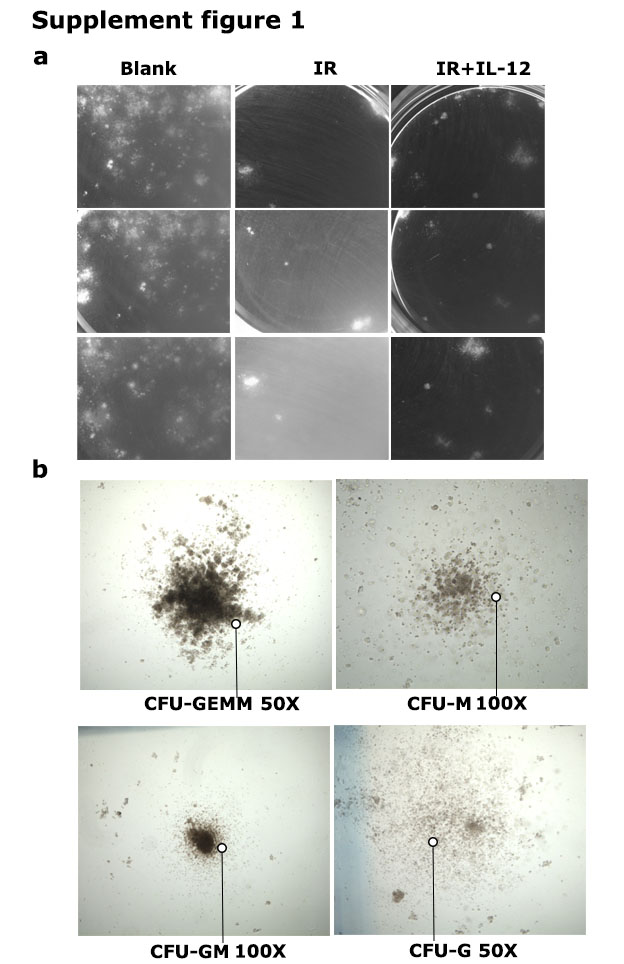

Supplement: Supplementary Figure 1 — IL-12 promotes hematopoietic stem and progenitor cells (HSPCs) colonies formation after irradiation. (a) HSPCs colonies without stain were taken photos under the light microscope. 2.5X. (b) Different types of colony-forming units (CFU) of granulocyte (CFU-G) and macrophage (CFU-M), CFU-granulocyte macrophage (CFU-GM), CFU-granulocyte, erythroid, macrophage, megakaryocyte (CFU-GEMM) were analyzed according to the technical manual. [file Image_1.tif]

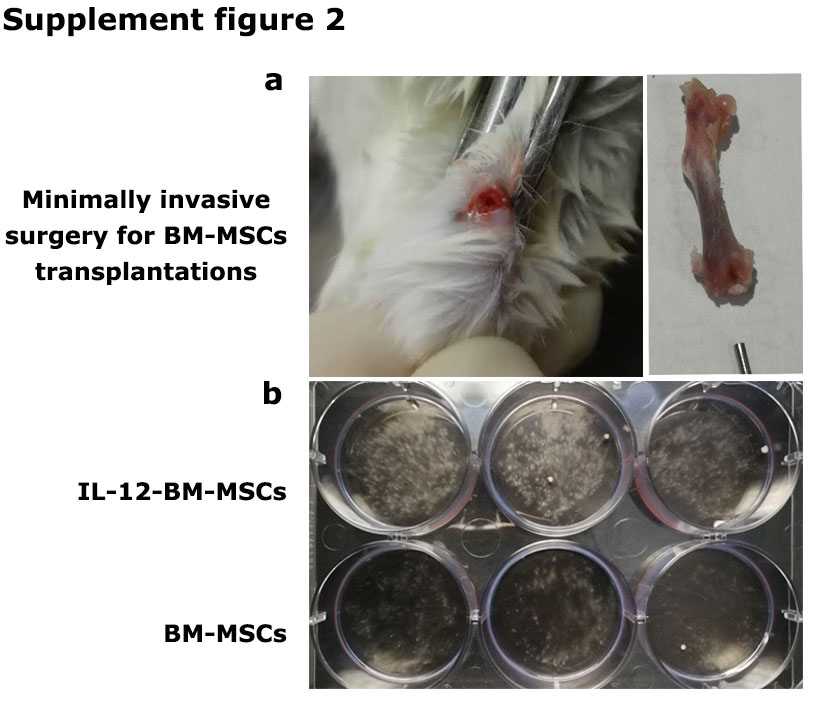

Supplement: Supplementary Figure 2 — The capacity of bone marrow colony-forming unity fibroblast (CFU-F) in two femurs of the same individual after transplantation of IL-12 treated bone marrow mesenchymal stem cells (BM-MSCs) (IL-12-BM-MSCs) and BM-MSCs (a) Minimally invasive transplantation at distal femurs of irradiated mice and (b) HSPCs colonies of the left femurs, invasive transplantation with BM-MSCs treated with IL-12 for 24 h, and right femurs, invasive transplantation with BM-MSCs treated without IL-12, of the same individual without staining. [file Image_2.tif]

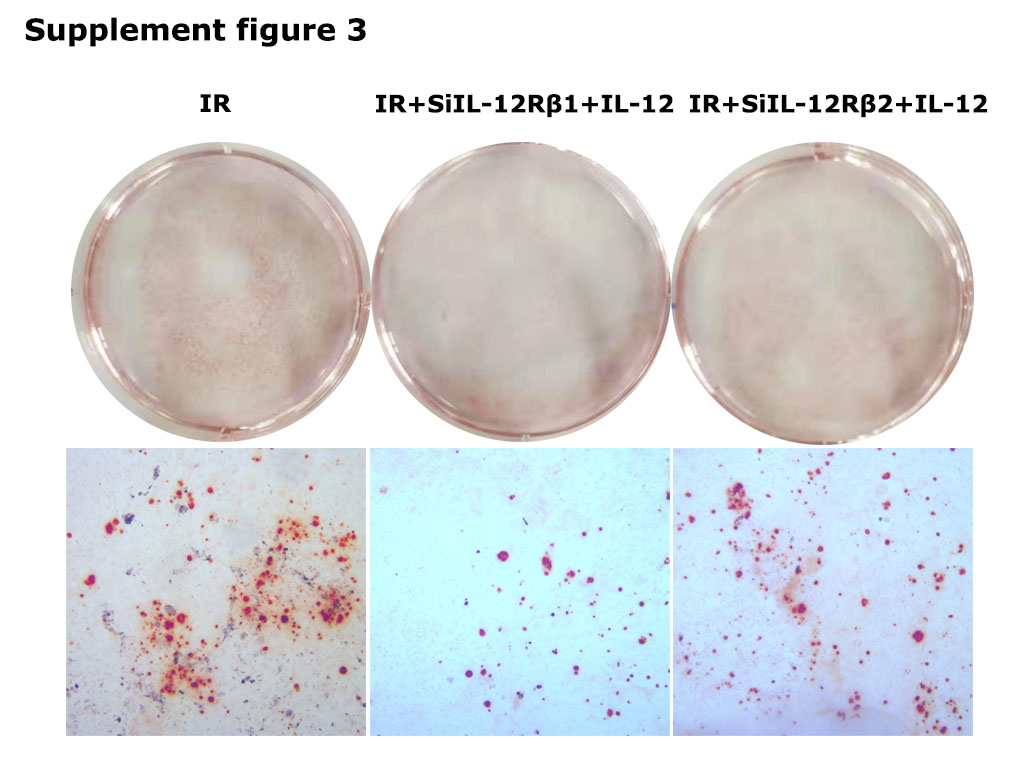

Supplement: Supplementary Figure 3 — The function of IL-12 receptors in the osteogenic process of BM-MSCs co-cultured with 0.2 ng/ml IL-12 in osteogenesis inducing medium after irradiation. Alizarin red S staining of calcium depositions in cells co-cultured IL-12 on day 1 after irradiation in osteogenesis inducing medium for 14 days. The magnifications are 200×. [file Image_3.tif]
